# Cultural Control of *Drosophila suzukii* in Small Fruit—Current and Pending Tactics in the U.S.

**DOI:** 10.3390/insects12020172

**Published:** 2021-02-17

**Authors:** Torsten Schöneberg, Margaret T. Lewis, Hannah J. Burrack, Matthew Grieshop, Rufus Isaacs, Dalila Rendon, Mary Rogers, Nikki Rothwell, Ashfaq A. Sial, Vaughn M. Walton, Kelly A. Hamby

**Affiliations:** 1 Department of Entomology, University of Maryland, College Park, MD 20742, USA; torschon@umd.edu (T.S.); mtlewis@umd.edu (M.T.L.); 2 Department of Entomology & Plant Pathology, North Carolina State University, Raleigh, NC 27695, USA; hjburrac@ncsu.edu; 3 Department of Entomology, Michigan State University, East Lansing, MI 48824, USA; grieshop@msu.edu (M.G.); isaacsr@msu.edu (R.I.); 4 Department of Horticulture, Oregon State University, Corvallis, OR 97331, USA; dalila.rendon@oregonstate.edu (D.R.); vaughn.walton@oregonstate.edu (V.M.W.); 5 Department of Horticultural Science, University of Minnesota, St. Paul, MN 55108, USA; roge0168@umn.edu; 6 Northwestern Michigan Horticultural Research Center, Michigan State University, Traverse City, MI 49684, USA; rothwel3@msu.edu; 7 Department of Entomology, University of Georgia, Athens, GA 30602, USA; ashsial@uga.edu

**Keywords:** exclusion, pruning, mulching, irrigation, microclimate, sanitation, postharvest, IPM, spotted-wing drosophila

## Abstract

**Simple Summary:**

Integrated Pest Management (IPM) is a science-based decision-making process that uses a variety of management approaches to increase farm profitability while protecting human health and the environment, with pesticides used only as a last resort. An important alternative to pesticides, cultural controls modify production practices and/or the crop environment to reduce pest populations and damage. This review presents the current state of knowledge and implementation of cultural controls to manage the invasive vinegar fly, spotted-wing drosophila, in U.S. small fruit crops. Spotted-wing drosophila causes direct damage by laying its eggs into ripening fruit. Because it reproduces quickly, uses a variety of cultivated and wild fruits, and is highly mobile, spotted-wing drosophila is difficult to manage. Developing effective and economic cultural controls to manage spotted-wing drosophila will help improve IPM programs.

**Abstract:**

Spotted-wing drosophila, *Drosophila suzukii* (Matsumura) (Diptera: Drosophilidae), a vinegar fly of Asian origin, has emerged as a devastating pest of small and stone fruits throughout the United States. Tolerance for larvae is extremely low in fresh market fruit, and management is primarily achieved through repeated applications of broad-spectrum insecticides. These applications are neither economically nor environmentally sustainable, and can limit markets due to insecticide residue restrictions, cause outbreaks of secondary pests, and select for insecticide resistance. Sustainable integrated pest management programs include cultural control tactics and various nonchemical approaches for reducing pest populations that may be useful for managing *D. suzukii.* This review describes the current state of knowledge and implementation for different cultural controls including preventative tactics such as crop selection and exclusion as well as strategies to reduce habitat favorability (pruning; mulching; irrigation), alter resource availability (harvest frequency; sanitation), and lower suitability of fruit postharvest (cooling; irradiation). Because climate, horticultural practices, crop, and market underlie the efficacy, feasibility, and affordability of cultural control tactics, the potential of these tactics for *D. suzukii* management is discussed across different production systems.

## 1. Introduction

Cultural controls that modify production practices and/or the crop environment can play an important role in limiting pest populations and damage [1,2]. These approaches reduce the availability and suitability of crop habitats for pests by limiting space and resources for feeding, mating, and egg laying, as well as removing shelter from natural enemies and adverse weather [3,4]. Within fruit production systems, cultural control practices are most commonly adopted to improve production and fruit quality rather than to manage pests [5], although they may provide additional benefits by affecting the crop environment and pest pressure [6,7,8]. Cultural controls can also reduce dependence on pesticides, which delays pesticide resistance, and they are critical for implementing sustainable integrated pest management (IPM) programs.

**Figure 1:**
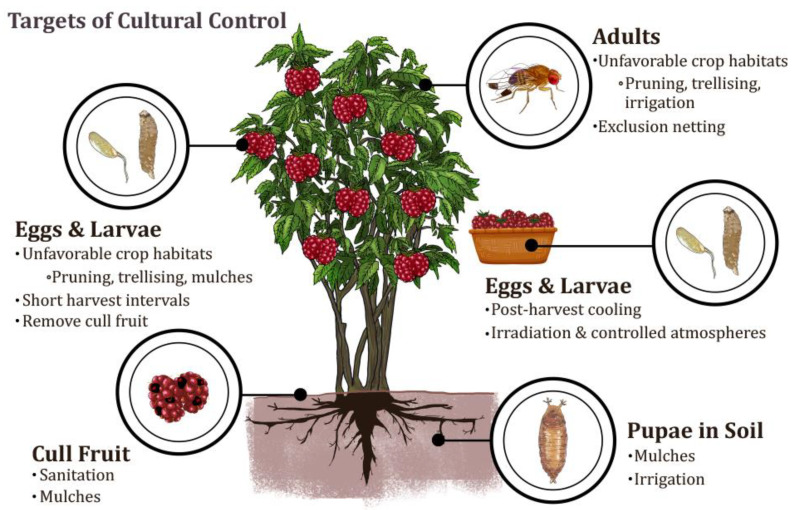
Life stages of *D. suzukii* targeted by cultural control practices. Figure Credit: Naiade Caparelli.

Since its arrival to the continental United States in 2008, *Drosophila suzukii* (Matsumura) (Diptera: Drosophilidae) has disrupted Integrated Pest Management (IPM) programs across major U.S. fruit-growing regions [9]. Although *D. suzukii* accepts a broad spectrum of hosts and ripeness, females often use their sawlike ovipositor to lay eggs into soft-skinned, ripening and ripe fruit including blueberries, caneberries, strawberries, and cherries [10,11,12,13,14]. Their short reproductive cycle and quick development allows populations to grow rapidly and makes management extremely difficult [15,16,17]. *Drosophila suzukii* has dramatically impacted the economics of small fruit production due to increased management costs, direct fruit damage, and the potential for contamination of the fruit with secondary pests and diseases [18,19,20,21,22,23]. In the absence of alternative management tactics, growers rely on broad-spectrum insecticides [19,24,25]. Implementing and developing cultural control techniques that target various *D. suzukii* life stages could help restore IPM programs for small fruit production (Figure 1).

In the U.S., nearly 34,000 farms produce small fruit on more than 300,000 acres [26], with production systems varying geographically and encountering regional and crop-specific challenges for *D. suzukii* management [5,27]. For example, the majority of fresh market small fruit is hand harvested, while fruit bound for processing markets is commonly mechanically harvested [5,28,29,30]. Mechanical harvesting reduces labor costs, but requires a large volume of ripe fruit on the plant at the same time, which extends the growing period and provides additional time for pests to damage fruit [31]. Achieving consistent, thorough harvests can also be challenging on small scale or you-pick fruit farms, where unharvested or cull fruit can act as a reservoir for *D. suzukii* populations. Ultimately, successful cultural control programs will vary depending on the production system (retail, export, you-pick), marketing strategy (quick frozen, juice, fresh), farm size, and regional climate. While there is no one-size-fits-all approach to cultural management of *D. suzukii*, some cultural tactics show broad promise and others may be useful in specific settings (Table 1).

Certain cultural control methods can be used irrespective of farm size, crop, or climate. For example, crops and cultivars that fruit early in the growing season are less vulnerable to infestation because *D. suzukii* populations build over the season [32,33,34]. Pruning is a routine procedure used to increase plant health and fruit quality [5] that may also contribute to *D. suzukii* management. Habitat suitability can be altered because *D. suzukii* is sensitive to extreme abiotic conditions [17,35,36,37,38]. However, the ability to sufficiently reduce habitat suitability is affected by the climate of the growing region; for example, tactics to reduce relative humidity can be more successful in arid regions. Cultural decisions made during the establishment of plantings can also influence pest pressure, and at-planting tactics such as mulching or trellising may be hard to modify once plants are established. Row orientation can help to increase sunlight interception (north–south rows vs. east–west rows) and air flow [5], which can affect the microclimate experienced by *D. suzukii*. Frequent harvesting and appropriate disposal of overripe or cull fruit reduces potential oviposition sites and keeps populations manageable during the season [39,40]. Although these sanitation measures may have potential for all production systems, they require a considerable amount of labor. Exclusion netting or plastic row covers also require careful consideration, as the initial investment can be high and amortization can take several years [41,42]. Ultimately, growers need research-based information to decide which cultural control methods are most economic, feasible, and least labor intensive for their production system.

Tremendous efforts have recently been made to evaluate cultural control strategies for *D. suzukii*. This article reviews recent literature on potential strategies, broadly categorizing cultural tactics into preventative approaches that reduce *D. suzukii* pressure, tactics that alter the within-crop microclimate, and tactics that reduce resource availability. We also discuss approaches to mitigate losses through postharvest management, followed by a case-study synthesis section. We conclude with an outlook into future research directions and approaches to increase the adoption of cultural controls for this pest.

## 2. Cultural Control Tactics

### 2.1. Preventative Tactics to Reduce Pressure

Minimizing pest damage is an overarching goal of IPM and includes strategies to create asynchrony between pest occurrence and fruit ripening as well as excluding pests from fruit to reduce infestation. For example, planting earlier ripening or less susceptible cultivars reduces the likelihood of *D. suzukii* infestation as populations build up during the latter portion of the season. Barricades that exclude pests from fruit can be effective and reduce the need for curative strategies later in the growing season, delaying insecticide applications or reducing the number required. Successfully implementing these tactics requires careful planning, regular sampling of fruit, and an initial financial investment that may limit their feasibility in certain production systems.

#### 2.1.1. Crop and Cultivar Selection

*Drosophila suzukii* is able to utilize a wide variety of cultivated and wild fruit. Within cultivated small fruit crops, susceptibility to *D. suzukii* infestation varies and preference may be affected by multiple factors including seasonality and phenology, fruit availability, fruit ripeness and maturity, chemical factors such as volatile organic compounds, fruit pH, soluble sugar content or acidity, and physical factors such as skin thickness, fruit firmness, and fruit texture. Ripe fruit is more susceptible to *D. suzukii* than unripe fruit [14,43,44]. Fruit softens and becomes sweeter as it ripens, and *D. suzukii* oviposition and larval development are positively correlated with °Brix (soluble sugars) and negatively correlated with fruit firmness [14,45,46]. These characteristics vary across crop types and varieties, and are also influenced by environment and production practices [47,48].

To date, few studies have focused on plant resistance to *D. suzukii.* In choice and no-choice laboratory bioassays, female *D. suzukii* exhibited oviposition preferences for certain blackberry and late-season blueberry cultivars. No differences in preference were observed among raspberry and wine grape cultivars [14], but separate field surveys in North Carolina reported variable larval infestation rates among different raspberry, blueberry, and blackberry cultivars [45]. These differences in cultivar susceptibility were attributed to variation in fruit firmness and penetration force between cultivars, with fewer eggs laid in firmer fruit [45]. The role of fruit firmness was corroborated in laboratory comparisons of raspberry cultivars [46]. Additional interactions between the fruit’s chemical and physical properties may also influence susceptibility. For example, strawberry cultivars that produce high levels of methyl anthranilate experience lower infestation levels, although oviposition was similar across cultivars [49]; this trend may reflect limited *D. suzukii* egg viability and larval development in cultivars that produced high levels of this compound [50]. Across blueberry cultivars, more eggs were laid and more *D. suzukii* developed in fruit as pH increased [14]. Laboratory studies using artificial substrates also suggest that *D. suzukii* females may make oviposition choices that strike a balance between fruit firmness and sugar content [45]. Differences in oviposition and larval development between Italian grape cultivars were driven by fruit texture including skin firmness, elasticity, and pulp consistency. *Drosophila suzukii* was better able to take advantage of cultivars with softer skin and pulp with lower elasticity, while chemical factors including pH, soluble solids and acidity did not influence oviposition and larval development [51]. Understanding the physical and chemical attributes that influence fruit susceptibility may help growers select tolerant cultivars and could also guide future breeding efforts.

Crops can be selected to phenologically escape peak pest populations. *Drosophila suzukii* populations fluctuate seasonally, with specific patterns varying across different climates and geographic areas [16,33,34,52]. In temperate regions, peak *D. suzukii* populations typically occur later in the growing season [32,53], so fruit that matures prior to these population peaks will be less impacted by *D. suzukii* in most years. For example, blueberry cultivars that ripened prior to 25 July in Rhode Island were not infested by *D. suzukii*, whereas later maturing varieties were more greatly impacted [54]. Primocane-bearing (fall-bearing, fruit on first year canes) raspberries produce ripe fruit later in the season and usually experience high *D. suzukii* pressure [34,45,55]. Therefore, selecting early maturing crops and varieties can be an important preventative approach. However, because seasonal phenologies of the pest and crop are dependent on local weather conditions and vary from year to year, early maturing varieties may occasionally suffer damage, especially in years where *D. suzukii* populations are less affected by winter conditions [56].

#### 2.1.2. Exclusion

Fruit growers are increasingly using protected culture practices to preserve crop yield and quality, reduce damage from abiotic factors, expand the growing season, and protect crops from pests while meeting consumer demand for high quality fruit and reduced pesticide applications [57]. Physical exclusion using fine mesh netting (1.0 × 0.6 mm in size or smaller) can be installed on structures (e.g., high tunnels) and has effectively reduced *D. suzukii* infestation in raspberries [42,58,59], blueberries [41,60,61], blackberries [62] and wine grapes [63] compared to uncovered open field plots.

The efficacy of physical exclusion depends on various factors, including *D. suzukii* pressure, host crop attractiveness, crop phenology, timing of installation, and grower management. High pest pressure primocane-bearing raspberry experiments show that exclusion netting can delay or reduce *D. suzukii* infestation and limit the need for pesticide applications [42,59]. However, later in the season *D. suzukii* populations may build under protected habitats, exceeding densities observed in open field locations [58] and necessitating additional management practices. To be effective, exclusion netting should be installed before *D. suzukii* becomes active. Flies may enter protected habitats via holes or gaps in the netting or when farm workers enter these structures; therefore, entryway vestibules may offer further protection and reduce colonization of protected habitats. Areas that frequently experience severe weather (e.g., high winds) that can damage the netting and structures may be less suitable for protected culture.

Other potential drawbacks of using fine mesh netting and protected habitats to exclude *D. suzukii* include modification of the microclimate, effects on yield and fruit quality, increased incidence of disease, insufficient crop pollination, reduced abundance of natural enemies, potential negative effects on agritourism, and plastic waste. Marketable fruit yields tend to be higher when fruit is protected under fine mesh netting [58,59,62] and fruit quality is comparable to open field plots [42,60,61]. Fine mesh netting and protected culture potentially reduce ventilation and increase air temperatures; however, air temperatures in these systems were similar to open field plots [42,58,59,60,61]. The initial investment costs of physical exclusion can be high, and fruit grown under fine mesh netting may show higher incidence of botrytis fruit rot and crumbly berry, indicating insufficient pollination [58,59]; thus, in crops where fruit ripening and flowering co-occur, supplemental pollination may be required to ensure adequate yield and crop quality.

### 2.2. Manipulating within Crop Microclimate

*Drosophila suzukii*’s narrow optimum temperature and humidity range (22–28 °C; ≥70% relative humidity [35,64]) may restrict the suitability of crop microhabitats. For example, exceeding optimal environmental conditions can cause habitat avoidance, reduced survival, oviposition and development, and damage reproductive organs [17,35,36,37,38]. To exploit *D. suzukii*’s sensitivity to abiotic conditions, management techniques such as pruning, trellising, mulching, and irrigation can be used to create less hospitable environments for both adults and larvae. Combining multiple strategies is likely to have the greatest effect on *D. suzukii* populations. These measures alone will not provide complete control; however, they may cause sublethal effects and alter adult activity patterns, complementing other tactics within *D. suzukii* IPM programs.

#### 2.2.1. Pruning and Trellising

Pruning is a routine procedure used to modify crop architecture, which improves plant health and vigor as well as fruit quality, yield, and production efficiency [5]. Pruning may also alter microclimate suitability for *D. suzukii* by opening the canopy, increasing airflow, and limiting shade and relative humidity [65]. Indeed, captures of adult *D. suzukii* in south-west Germany were up to two times higher inside dense, minimally pruned grape canopies compared to the standard pruning and trellising system [66], suggesting that pruning reduces adult activity and/or larval infestation. Pruning may be particularly effective in floricane-bearing (summer-bearing, fruit on second year canes) caneberries, where fruit-bearing floricanes and newly emerged vegetative primocanes grow together to produce a dense, shady canopy. High levels (25–50% canopy reduction) of pruning within caneberries reduced *D. suzukii* infestation up to 80% in California compared to denser canopies; however, low (8–33%) or no reduction was observed in more humid climates [55]. Likewise, lower fruit infestation was observed in highly pruned blackberry canopies in North Carolina compared with minimally pruned blackberry canopies [67]. In contrast, heavy pruning had no effect on *D. suzukii* infestation rates in grapes [66] and minimal effect in blueberries [55]. Changes in infestation likely reflect differences in crop microclimate, particularly lower relative humidity. Temperature minimally varied between pruning treatments, with slightly higher temperatures (0.0–1.7 °C) observed in highly pruned caneberries throughout the U.S. [55] and in less dense grape canopies (0.1–0.9 °C) in Germany [66].

In caneberry systems, trellising may be an additional tactic that could be integrated with pruning to further open the canopy and improve light penetration and air circulation. Various trellising options are available for raspberry and blackberry production, including the I-trellis, T-trellis, V-trellis, and rotating (swinging) cross arm trellis [5,28]. The I-trellis is a commonly used design that consists of either a single wire or two separated wires secured to large wooden posts [5,28]; though relatively inexpensive to install, the I-trellis forms a dense hedgerow canopy that may create optimal microclimates for *D. suzukii*. In contrast, the V-trellis is built with two metal posts that are set at opposite angles to form a V shape. This design allows for increased air circulation (lowers relative humidity) and light penetration (increases temperature) compared with the I-trellis [5,68] and may be more suitable for *D. suzukii* management. Rotating cross arm trellises that pivot so fruit is located only on the shaded side of the row require a higher financial investment and more intensive labor and maintenance, but facilitate harvest and minimize sun damage to the fruit. However, further research is needed to understand how these trellises affect the microclimate and suitability for *D. suzukii*.

Pruning and trellising are constrained by crop seasonal phenology and typically need to be conducted early in the season when the plants are dormant. These tactics also increase production costs due to the additional labor and materials that their installation requires. However, the benefits that pruning and trellising provide may offset these limitations. More open canopies improve harvest efficiency and thus help with sanitation measures by reducing oviposition sites [69]. Additionally, open canopies receive better spray coverage [70,71]. In conclusion, pruning and trellising can be important components of an IPM strategy, rendering habitats less suitable for *D. suzukii* while maintaining yield and fruit quality.

#### 2.2.2. Mulching and Ground Management

*Drosophila suzukii* larvae typically leave the fruit to pupate in the top 0.5 cm layer of the soil [72]. This is facilitated by fruit falling from the canopy to the ground (60%–80% of infested fruit fall), and 82%–93% of the larvae pupate in the soil [73]. During the growing season, most of the *D. suzukii* population is comprised of immature stages inside fruit [16] that are mostly impervious to insecticides; therefore, cultural controls that target pupae and larvae will complement existing IPM programs.

Different types of mulches can modify ground and canopy climate (temperature and relative humidity), potentially creating an environment less suitable for *D. suzukii* development. For example, black plastic mulches are increasingly used for weed control as well as to increase soil temperature and reduce humidity [74,75]. Depending on the type of material used, mulches can also modify above-ground air temperatures, photosynthetic active radiation, and light intensity [76,77]. To understand how mulches affect *D. suzukii*, a recent study examined the influence of black woven weedmats on temperature, relative humidity, and immature survival in blueberries across different regions in the U.S. [78]. Two study regions (Maryland and Michigan) used field sites with young plants and small canopies; at these sites, the plots with weedmats had higher ground temperatures and lower levels of *D. suzukii* larval survivorship or natural infestation, a difference that could reflect factors such as increased solar radiation, increased light penetration, or reduced shade in the younger plantings, though further investigation is needed [78]. However, weedmats did not significantly affect relative humidity, ground temperature or *D. suzukii* larval and pupal survivorship in the other regions (Georgia, Minnesota, Oregon) studied. Weedmats can also provide an effective barrier preventing larvae from burying into the ground to reach more suitable pupation microhabitats; *D. suzukii* larvae and pupae have a significantly lower survival rate above the ground than below the ground [78]. Incorporating mulches or weedmats can also provide various horticultural benefits, while acting in conjunction with other *D. suzukii* management methods.

In addition to mulching, soil tillage has been explored as a method to control *D. suzukii*; however, tilling the soil between organic blackberry rows did not significantly modify adult captures or fruit infestation [79]. Ground management tactics such as mulching and mowing are one of the most predominant preventative measures used against *D. suzukii* in Swiss grape crops [80], and their effectiveness in other crop systems and regions needs further study.

#### 2.2.3. Irrigation

Irrigation methods impact arthropod pest populations in multiple crops [81,82] and may be exploited for *D. suzukii* management. Changing the amount of water delivered or the type of irrigation system used can modify temperature and/or relative humidity within the crop. In Oregon blueberries, above and below ground temperatures were not altered by drip or overhead sprinkler irrigation, but relative humidity was lower in drip-irrigated plots [83]. This correlated with reduced *D. suzukii* pupal survivorship, though the survival of larvae inside infested blueberries was not affected [83]. This suggests that larvae are protected from desiccation while developing inside the fruit, but become more susceptible and succumb to desiccation after leaving the fruit to pupate. Irrigation may also affect adult activity and/or oviposition behavior. In choice bioassays, adult *D. suzukii* avoided low humidity conditions [36] and oviposition rates were highest in the center of blueberry bushes, a trend that positively correlates with relative humidity [84]. However, a recent study in blueberries found that fly activity (the number of flies visually counted flying in or on bushes) did not differ between irrigated and nonirrigated plots treated with zeta-cypermethrin. This pattern remained similar before, during, and after an irrigation period and regardless of pesticide application [85].

In addition to altering the crop microclimate, irrigation (or simulated rainfall) can remove insecticide residues, potentially affecting efficacy, with effects sometimes observed multiple days after exposure. In semi-field assays, flies exposed to blueberry fruit and shoots collected one day after zeta-cypermethrin treatment experienced lower mortality when the plots were irrigated for 60 min compared to flies that were exposed to fruit and shoots from nonirrigated plots; no difference in survival occurred in plots irrigated only for 15 min [85]. In blueberries, all levels of simulated rainfall (overhead irrigation) increased adult fly survival relative to the no-rainfall controls when flies were exposed to insecticide treated shoots one day after treatment, irrespective of the insecticide applied [86]. Comparably, in a semi-field study where cherry shoots treated with six different insecticides were exposed to a rainfall simulator with two different levels of rainfall, adult and offspring survival increased in both rainfall treatments relative to the no-rainfall control [87].

Considering the interactions among irrigation, environmental conditions, and pesticide efficacy, overhead irrigation provides a more suitable environment for *D. suzukii* because it can reduce pesticide efficacy and increase relative humidity. Irrigation methods that do not spray water on the canopy (e.g., drip or flood) are expected to reduce *D. suzukii* pest pressure as well as conserve water compared to overhead applications.

### 2.3. Harvest Management

Despite exhibiting a preference for healthy, undamaged fruit, *D. suzukii* will use alternative hosts when intact fruit are less available [10]. This includes damaged or decaying fruit, fruit wastes, mushrooms, and even animal feces [10,14,88,89,90]. In addition, *D. suzukii* has a high dispersal capacity [91,92], allowing it to exploit diverse habitats where unmanaged fruiting plants grow [93]. Accordingly, diversified agricultural landscapes allow *D. suzukii* populations to develop and build on early ripening crops (e.g., strawberries and tart cherries) and then readily move to mid- or late-season crops (e.g., blueberries, raspberries, grapes). Reducing alternate host resources within a farm therefore may help reduce or delay *D. suzukii* population growth.

Harvest schedules and sanitation practices can affect the amount of host resources available to *D. suzukii*. Shortening the harvest frequency from every three days to every one or two days reduced *D. suzukii* infestation up to 60% in raspberries [40]; the feasibility of this practice depends on the type of operation and the crops produced. Waste fruits are common throughout the harvest process, including culls in the field, culls from processing lines, and leftover fruit in the canopy or on the planting floor. These can all provide resources for *D. suzukii*, so they should be minimized to limit population development.

For hand-picked fruit, training harvest teams to reject and separately collect externally damaged or soft fruit can minimize postharvest larval infestation and help ensure that fruit meets fresh-market standards. Processed fruit is often harvested mechanically, with fruit removed regardless of quality. However, soft-sorting machines and other postharvest quality control processing steps can remove infested fruit from the final product. Early harvest can reduce infestation by *D. suzukii* in lowbush blueberry, but early harvest may also increase the number of unripe fruit harvested and thereby reduce yield, so the economic benefits and limitations of this strategy must be balanced [94].

### 2.4. Postharvest Sanitation

The management of cull fruit wastes is important for sanitation to reduce populations of *D. suzukii*. Bagging and subsequent solarization can provide a near 100% reduction in fly emergence but is best suited to relatively small quantities of fruit [40]. Evaluations of burial suggest that shallow burial is not effective at farm scales [95] and that burial depths must be at least 24 cm to reduce emergence by 95% [39]. Composting fruit wastes with different feedstocks has also shown promise. Although laboratory experiments indicate that the combination of apple pomace (cider pressings) with high carbon vegetable feedstocks (leaves and sawdust) only affected *D. suzukii* reproduction potential by dilution (e.g., the addition of 20% feedstock led to a 20% reduction in F1 generation), relatively small amounts of high nitrogen chicken manure reduced survival by 80–100% [96]. Similar results were observed in a subsequent field experiment for larger quantities of pomace containing *D. suzukii* and other *Drosophila* spp. [96].

Crushing fruit wastes and adding black soldier fly *Hermetia illucens* (L.) (Diptera: Stratiomyidae) to fruit waste are two additional postharvest sanitation approaches that have been preliminarily explored but have yet to be rigorously evaluated. Crushing of infested Montmorency tart cherries on the orchard floor using a golf cart eliminated reproduction of *D. suzukii* in crushed fruit within 72 h, while reproduction continued for at least nine days in uncrushed fruit (N. Rothwell, unpublished). Black soldier fly reduces or eliminates pest flies in animal manures [97,98], and preliminary laboratory trials suggest that the addition of early instar black soldier fly to small volumes of fruit waste can eliminate *D. suzukii* reproduction, either by influencing oviposition decisions or by direct competition with or predation by developing black soldier flies (M. Grieshop et al. unpublished).

### 2.5. Reducing Suitability of Postharvest Fruit

Modifying the fruit storage environment postharvest is an effective and common strategy to manage many internally feeding insects. Depending on the crop, these modifications can include reducing storage temperature or short-term exposure to high temperatures, low oxygen atmospheres, or radiation. These latter techniques are used primarily for quarantine purposes to prevent the spread of invasive species; cold storage, atmospheric manipulation and irradiation have all been explored as potential nonchemical postharvest control tactics for *D. suzukii.*

#### 2.5.1. Cooling

Both cold and heat treatments have been developed for postharvest insect control, and as with other postharvest management strategies, temperature modification is most well studied with respect to quarantine pests [99]. As a group, Drosophilidae are believed to have originated in the Afro-tropics, and chill-susceptibility is considered their ancestral state [100]. Cold tolerance has been extensively studied within *D. melanogaster* and has been demonstrated to be variable among populations of this species and malleable to a certain extent based on conditioning [101].

*Drosophila suzukii* egg and larval mortality increases when exposed to cold temperatures within artificial diet or fruit [102,103]. While *D. suzukii* also appear to increase their cold hardiness following gradual acclimatization either in the laboratory [104,105,106] or under natural fall and winter conditions [107,108], they cannot adapt when larvae in harvested fruit are placed directly from spring or summer growing conditions into low temperature storage. Temperatures lower than 5 °C slow or arrest immature *D. suzukii* development for up to three days, and storage temperatures of 1.1 °C and lower for three days or more result in significant mortality [102,103]. Eggs and first instar larvae appear consistently susceptible to cold storage while second and third instars are more variable in their tolerance [109]. Combined with frequent, thorough harvest to reduce immature development time in fruit prior to picking, cold storage can provide growers with a means to maintain marketability even if a field infestation is detected. Maintaining constant cold storage throughout the fruit supply chain, from grower to consumer, not only increases the shelf life of fruits but also reduces the likelihood of infestation resulting in damaged or unmarketable fruit.

#### 2.5.2. Irradiation and Quarantine Management

Treatment of fruit with high doses of radiation is most often used in quarantine programs for invasive pests to prevent their export. Effective doses vary by species, but guidance has been developed at the order and family level for some groups [110]. While irradiation is not considered a chemical treatment, its use in postharvest insect control is not universally accepted in all countries [110], and in the U.S., irradiated food products do not meet criteria for organic certification [111]. Because irradiation requires specialized equipment and is potentially risky to users, it is unlikely to be utilized for general *D. suzukii* postharvest control. However, irradiation may provide control of *D. suzukii* in export or quarantine situations.

Developing irradiation protocols for postharvest fruit treatments is considered easier than developing temperature or modified atmosphere treatments, because radiation levels that kill insects typically do not damage fruit [110]. Guidance levels for *D. suzukii* quarantine treatments have recently been developed. Different experiments have suggested slightly different doses and exposure methods for use against *D. suzukii* in fruit. In one series of experiments, adult emergence was completely prevented when first and second instar larvae were exposed to a dose of 40 Gray (Gy) [112]. Pupae, however, were more tolerant to radiation and required a dose of 80 Gy to prevent adult emergence [112]. In other experiments, adult emergence was not completely inhibited, even at higher doses, but all surviving adults were sterile following exposure to 100 Gy [113]. Radiation sources also differed in their efficiency against *D. suzukii* with electron beam radiation resulting in higher pupariation compared with gamma irradiation, although both methods provide potentially acceptable quarantine level control [114].

Modified or controlled atmospheres which create low oxygen environments, and therefore limit the respiration of insects within fruit [115], may also be used in postharvest pest control. They can be used alone or in combination with other postharvest treatments, but for *D. suzukii*, they have only been assessed in combination with irradiation [116].

## 3. Adoption of Cultural Controls in IPM Programs

The invasion by *D. suzukii* has severely challenged cherry and berry IPM programs, and in some cases farms have adapted by growing other crops or by making other changes to their marketing and production strategies. The following specific perspectives represent situations where cultural controls were successfully adopted, and we acknowledge not all growers were in a similar position.

### 3.1. Blueberries—Pacific Northwest U.S.

Increased global production continues to tighten markets and shape production practices in Oregon and Washington (the Pacific Northwest, PNW). In the past, PNW growers were able to help fill the national and international supply gap during the late portion of the season, resulting in relatively high value crops from late August into September. The ever-increasing supply of blueberries worldwide has, however, caused these production regions to segregate themselves from other markets by focusing on crop quality. During the past 10 years, PNW blueberry production has begun to target high value fresh and export blueberry markets (D. Brazelton, personal communication), with an increasing portion of fresh blueberries produced in this region. During 2016 and 2018, 63% and 72% of all fresh blueberries in the U.S. were produced in the PNW, respectively. In British Columbia, Canada as well as Oregon and Washington, U.S., the majority of blueberries are organically produced. This market is driven by a price premium of up to two times higher than conventionally produced fruit. Producers seek this premium largely because of increasing capital and land costs, the cost of labor, and large volumes of crop produced by long-standing plantings of economically marginal cultivars.

The PNW benefits from relatively high yields, making premium (organic) fruit production viable. Overall, western production regions receive relatively little precipitation during the growing and crop ripening period [117]. These relatively arid, hot conditions are less suitable for *D. suzukii* survival and reproduction [83,118]. In addition, cold winters combined with few suitable microhabitats result in reduced overwintering survival and relatively narrow *D. suzukii* population bottlenecks in Eastern Washington compared to other U.S. production regions. Because this production system is high value, growers can use a relatively large amount of resources on cultural practices. Growers have adopted weed fabric and drip irrigation systems within their production system to decrease labor demands, increase crop productivity, reduce weed management costs and manage *D. suzukii* pest populations. Weed fabric is used in conjunction with drip irrigation placed directly underneath the fabric to decrease water use and weed management costs. The use of weed fabric and drip irrigation can also reduce *D. suzukii* pest pressure by reducing access to pupation sites and habitat favorability. Pruning choices are driven by growers wanting to produce a crop of premium value (D. Brazelton, personal communication) [119], though they may also reduce *D. suzukii* pressure [55]. Therefore, pruning can provide multiple benefits. Currently few growers make use of protective coverings as pesticide application efficacy is not hampered by the minimal summer precipitation, unlike other production regions [59]. However, some growers have diversified their production practices to stretch the production season into October, to supply fresh, local fruit to local metropolitan regions (D. Brazelton, personal communication [117]). Some of these practices include the use of tunnel systems, and late cultivars. PNW growers continue to fund investigations into new tools to decrease input costs; including cultural practices as well as behavioral tools such as deterrents and behavioral modifiers [120,121].

### 3.2. Caneberries—Eastern U.S.

Cultural control methods have great potential to reduce the need for chemical control of *D. suzukii* in caneberries. Of the available options, pruning, netting, harvest management, and cold storage are the most widely used and adaptable. Some farms in the eastern U.S. including The Berry Patch farm in New York [122] and Nourse Farms in Massachusetts have combined technical expertise in growing berry plants with a commitment to optimize nonchemical approaches before using insecticides.

Netting has minimized pest pressure in demonstration plots at The Berry Patch, and the owners of this farm have had great success with using this at their you-pick plantings of raspberries and blueberries. This has been installed on lightweight PVC support hoops, and it can also be retrofitted to high tunnels. High tunnels are increasingly popular for berry production, and although growers might be concerned with the initial investment, Nate Nourse who worked with them at Nourse Farms from 2011–2017 reports a return on the investment within 3–4 years (N. Nourse, personal communication). Tunnels also lower disease rates, reduce bird predation, and provide shade that improves fruit quality. These benefits may also be improved by the installation of exclusion netting for reducing *D. suzukii* immigration. However, with concerns about high temperatures affecting plant growth in regions where summer heat may be an issue, growers can install fans for heat removal or roll up the sides of the tunnels. These actions can also be automated.

One newer aspect of raspberry production that has allowed for increased yields is double cropping. This works well with the newer cultivars; with appropriate pruning of field grown plants, a portion of the crop can be adapted to ripen in summer before *D. suzukii* populations increase. Potted plants can be held at controlled temperatures and later moved to fields or high tunnels to allow for flexibility of growth and fruiting to push cultivar ripening into times of the season when *D. suzukii* pressure is lower. With these adjustments in the ripening period and production approach, growers may be able to harvest with fewer people, keep pickers busy from July to November, and harvest more berries per acre (N. Nourse, personal communication). This dovetails with needing to pick every day to maintain fruit quality and break the *D. suzukii* life cycle. After harvest, berries are placed in a cold chain at 0.6 °C (33 °F) where they can maintain quality for 3–5 days. Some farms may have a mix of protected and open field production, and at Nourse Farms they divide staff between these, allowing for picking berries in dry conditions, either in outside fields when it is not raining or in protected (high tunnel) fields when raining. By implementing these approaches, Mr. Nourse reports being able to grade 99.9% of the harvest as perfect berries in half pint clamshells. This required increased staffing to ensure that nothing went wrong in the production cycle, and to manage the complexities of the new system.

Overall, the experiences of The Berry Patch and Nourse Farms adjusting to *D. suzukii* invasion highlights that combining new cultivars, modern crop management, netting or high tunnels, adjusted harvest schedules, and postharvest cooling can greatly increase yields and fruit quality under commercial production conditions despite the challenges created by *D. suzukii*. This shows the effectiveness of cultural controls when they are combined in an integrated system, allowing for significant reduction in the need for insecticides and allowing biological control for other pests to flourish. Cost-share programs that allow growers to purchase high tunnels, or to start small by netting a portion of a farm, can help farms gain experience with this approach and see the benefits for themselves.

## 4. Future Directions

Developing effective and economic cultural control methods to manage *D. suzukii* will fill an important gap in existing IPM programs. In recent years, there has been a strong push to advance various cultural options for fruit production. However, tactics vary in their feasibility depending on farm size and marketing strategy. For example, exclusion netting systems require high initial investment in labor and materials. Given the costs associated with purchasing, installing, and properly disposing of materials, these tactics may be more suitable on farms with smaller scale fruit production or in operations that emphasize producing high value fruit. Likewise, additional pruning costs could be justified with concomitant increases to fruit quality, which may be less compatible with mechanical harvest and processing markets. Shortened harvest intervals and the removal of unmarketable or leftover fruit is usually not practical for you-pick farms that rely on consumers to harvest the majority of fruit and may not have the budget to train harvest crews or perform additional harvests. In some instances, the benefits of cultural control tactics extend beyond *D. suzukii* control, facilitating their adoption. For example, using mulches to target this pest may not always be economical, but the weed management benefits can offset limitations. Irrigation management can be a useful option in the arid areas of the U.S. west coast, with the additional benefit of potentially reducing water use. However, operations must invest in new drip irrigation materials if they currently rely on other irrigation systems. In addition to the initial input costs, the disposal of cultural control materials can also be challenging. For example, netting and plastic weedmat mulches become plastic waste [123]. Incentive programs and regulations on plastic waste disposal vary across the U.S. [124,125] and depending on the production region and the type of plastic used, recycling may be difficult [126] and these materials often biodegrade slowly [127]. Work addressing the financial and logistical barriers to the implementation of cultural control strategies, with systems-based cost benefit analyses, is necessary to develop and integrate economically viable cultural tactics into future IPM programs for this pest.

Several key knowledge gaps should be addressed to facilitate further integration of *D. suzukii* cultural controls into commercial production. In depth economic analysis of the individual tactics is needed across a variety of production systems; this will help ensure that the additional costs provide tangible benefits in terms of *D. suzukii* control, production goals, and farm profit. Additionally, many of the cultural control tactics discussed in this review have been tested individually. However, these approaches may have synergistic effects with other management tactics and should be studied in combination to develop the most effective IPM programs for specific farming scenarios. It will also be important to continue testing these tactics in collaboration with commercial fruit growers, as this will allow for further evaluation of feasibility and effectiveness across different farming operations. Such trials, in combination with a demonstrated environmental, social and economic benefit, will encourage grower adoption.

## Figures and Tables

**Table 1:** Cultural control practices used to manage *Drosophila suzukii* in blueberries and caneberries in the U.S. Table Credit: Naiade Caparelli.

Crop	Production System	Preventative Tactics	Unfavorable Crop Microclimate▲	Harvest Management	Post-Harvest Management
**Blueberry** 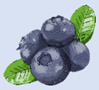	Fresh-Market Retail	Early season cultivars Exclusion netting 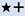	Pruning Weedmat mulch 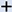 Drip irrigation	Harvest by hand More frequent harvest & remove cull fruit Training harvest team to sort fruit	Cold storage Irradiation■ Controlled atmosphere 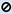 Cull fruit management
	Processed	Early season cultivars Exclusion netting 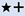	Pruning Weedmat mulch 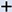 Drip irrigation	Mechanical harvesting Sorting machine	Cold storage Cull fruit management
	You-Pick	Early season cultivars Exclusion netting 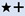	Pruning Weedmat mulch 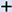 Drip irrigation	Consumer harvested	Educate consumers about cold storage
**Caneberries** 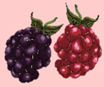	Fresh-Market Retail	Early season cultivars Exclusion netting 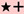	Pruning & trellising Weedmat mulch 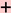 Drip irrigation	Harvest by hand More frequent harvest & remove cull fruit Training harvest team to sort fruit	Cold storage Irradiation■ Controlled atmosphere 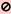 Cull fruit management
	Processed	Early season cultivars Exclusion netting 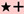	Pruning & trellising Weedmat mulch 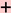 Drip irrigation	Mechanical harvesting Sorting machine	Cold storage Cull fruit management
	You-Pick	Early season cultivars Exclusion netting 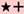	Pruning & trellising Weedmat mulch 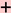 Drip irrigation	Consumer harvested Canopy management to improve efficiency of harvest	Educate consumers about cold storage

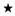
 Amortization, likelihood of in-season weather damage, impact on agritourism, and supplemental pollination should be considered. 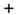 Can contribute to plastic waste and be difficult to recycle and break down. ▲ Most effective in regions that are arid during the growing season. ■ Export quarantine tactic. 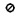 Not yet tested as a standalone technique.
